# Effect of primary tumor volume on survival of concurrent chemoradiotherapy in stage IV non‐small cell lung cancer

**DOI:** 10.1002/cam4.70221

**Published:** 2024-09-16

**Authors:** Xiaxia Chen, Wei Zhang, Lan Luo, Shimei Fu, Dongdong Cao, Shengfa Su, Qingsong Li, Wengang Yang, Yichao Geng, Bing Lu, Weiwei Ouyang

**Affiliations:** ^1^ Department of Oncology The Affiliated Hospital of Guizhou Medical University and The Affiliated Cancer Hospital of Guizhou Medical University Guiyang Guizhou China; ^2^ Department of Oncology Guizhou Hospital of the First Affiliated Hospital of Sun Yat‐sen University Guiyang China

**Keywords:** concurrent chemoradiotherapy, non‐small cell lung cancer, stage IV, survival, three‐dimensional radiotherapy

## Abstract

**Objective:**

To explore the survival effect of thoracic gross tumor volume (GTV) in three‐dimensional (3D) radiotherapy for stage IV non‐small cell lung cancer (NSCLC).

**Methods:**

The data cases were obtained from a single‐center retrospective analysis. From May. From 2008 to August 2018, 377 treatment criteria were enrolled. GTV was defined as the volume of the primary lesion and the hilus as well as the mediastinal metastatic lymph node. Chemotherapy was a platinum‐based combined regimen of two drugs. The number of median chemotherapy cycles was 4 (2–6), and the cut‐off value of the planning target volume (PTV) dose of the primary tumor was 63 Gy (30–76.5 Gy). The cut‐off value of GTV volume was 150 cm^3^ (5.83–3535.20 cm^3^).

**Results:**

The survival rate of patients with GTV <150 cm^3^ is better than patients with GTV ≥150 cm^3^. Multivariate Cox regression analyses suggested that peripheral lung cancer, radiation dose ≥63 Gy, GTV <150 cm^3^, 4–6 cycles of chemotherapy, and CR + PR are good prognostic factors for patients with stage IV non‐small cell lung cancer. The survival rate of patients with GTV <150 cm^3^ was longer than patients with ≥150 cm^3^ when they underwent 2 to 3 cycles of chemotherapy concurrent 3D radiotherapy (*p* < 0.05). When performing 4 to 6 cycles of chemotherapy concurrent 3D radiotherapy, there was no significant difference between <150 cm^3^ and ≥150 cm^3^.

**Conclusions:**

The volume of stage IV NSCLC primary tumor can affect the survival of patients. Appropriate treatment methods can be opted by considering the volume of tumors to extend patients' lifetime to the utmost.

## 1 INTRODUCTION

More than 50% of patients are diagnosed with stage IV NSCLC,^1^, ^2^, ^3^ and the 5‐year overall survival (OS) of NSCLC is still only 19%.^4^ IV NSCLC patients with EGFR gene‐sensitive mutations, ALK gene or ROS1 gene fusion can be treated with first‐line targeted drugs.^5^, ^6^, ^7^, ^8^ Immunotherapy drugs such as pembrolizumab monoclonal antibodies can be used for patients with PD‐L1 TPS ≥1%.^9^ Stage IV NSCLC patients without a driver gene is mainly treated by platinum‐based systemic chemotherapy, and the median survival of patients is 8–10 months.^10^ For the past 10–15 years, the efficacy of chemotherapy has reached a plateau.^11^ Systemic chemotherapy combined with other modalities may produce better results. Single‐center prospective clinical studies in our center and other retrospective studies have shown that phase IV NSCLC with three‐dimensional (3D) chest radiotherapy during chemotherapy can achieve encouraging clinical effects.^12^, ^13^, ^14^, ^15^

Some previous studies have found that the volume of the primary tumor is related to the survival of patients with stages I‐III NSCLC. The prognosis of patients with large‐volume primary tumor NSCLC is significantly worse than that of patients with small‐volume primary tumor and prescription dose can achieve long‐term survival.^16^, ^17^, ^18^ Jeffrey^19^ conducted a study on 207 patients with NSCLC who received 3D conformal radiotherapy. The results suggested that the primary tumor gross volume (GTV) delineated by the CT scan was closely related to the overall survival rate, tumor‐specific survival rate, and local control rate and was an independent prognostic factor. Therefore, the hypothesis based on the biological and clinical perspectives that the tumor volume significantly affects the outcome of radiotherapy was proposed. Dubben proposed that tumor volume can predict the outcome of radiation therapy more accurately than tumor size, location, or stage does.^20^

Regarding the survival effect of primary tumor volume in stage IV NSCLC with metastatic tumors, Ouyang et al. conducted a multi‐factor analysis of the results in a single‐center prospective study and found that the primary tumor volume <175 cm^3^ is independent factor for prognosis in stage IV NSCLC.^15^ Su et al. conducted other retrospective analyses of 93 patients with stage IV NSCLC and found that for stage IV NSCLC patients with larger GTV (≥170 cm^3^) or single metastatic lesions, OS in patients receiving ≥63 Gy group was significantly better than that in <63 Gy group. However, for patients with smaller tumors (GTV <170 cm^3^), there was no significant difference in survival between the ≥63 Gy group and the <63 Gy group.^13^ Therefore, the primary tumor volume of IV NSCLC is closely related to the overall survival rate and local control rate. Previous studies have found the effect of primary tumor volume on the survival of stage IV NSCLC through single or multiple factors, however, they had not provided stratified analyses of the effect of primary tumor volume on survival based on IV NSCLC with different clinical characteristics and concurrent chemoradiotherapy modalities. Therefore, we analyzed the survival effect of primary tumor volume of IV NSCLC who received at least 30 Gy 3D thoracic radiotherapy during the same period of chemotherapy.

## 2 MATERIALS AND METHODS

### 2.1 Enrolment criteria and patients selection

This retrospective study was approved by the Ethics Committee of Guizhou Cancer Hospital. Inclusion criteria: IV NSCLC (AJCC2002 staging) diagnosed by pathology or cytology treatment‐naive patients, aged 18 to 80 years, physical condition score karnofsky performance status (KPS) ≥ 70, patients did not undergo genetic testing or refused genetic testing, number of metastatic organs ≤5, It is expected to complete at least 2 cycles of chemotherapy, and 4–6 cycles to complete systemic chemotherapy without receiving maintenance treatment. Primary lung tumors and positive lymph nodes in the drainage area received at least 30 Gy of radiation. Three hundred and seventy‐seven patients were enrolled between May 2008 and August 2018, and the patients in the entire group were from 22 to 80 years old (median 57). There were 282 males and 95 females, and the ratio of male to female patients was around 2.97: 1. Pathological types include squamous cell carcinoma, adenocarcinoma, and other types of NSCLC. The GTV is defined as the sum of the volume of the primary lesion and the positive hilus as well as the mediastinal metastatic lymph node. The volume size is 5.83–3535.20 cm^3^, and the median volume is 190.08 cm^3^. Major distant metastatic organs include bones, brain, lung, and adrenal.

### 2.2 Chemotherapy regime

We propose a platinum‐based two‐drug combination chemotherapy regimen. The platinum drugs and the dosage are arranged as follows, paclitaxel (T) 140 to 170 mg/m^2^ or docetaxel (D) 60 to 75 mg/m^2^ divided into intravenous infusion on the first day, cisplatin (P) 80 mg/m^2^ or carboplatin (C) based on AUC = 5 and individualizing within 300–350 mg/m^2^ range at the second day, vinorelbine (N) 25 mg/m^2^ at the first day and the eighth day. Pemetrexed (P) 500 mg/m^2^ on the first day with cisplatin or carboplatin on the second day.

In 377 cases, the TP or TC scheme accounted for 10.9% and 0.3%, respectively. The ratio of DP or DC was 58.4% and 5.0%, respectively. The ratio of PP or PC was 15.9% and 1.6%, respectively. NP and other programs accounted for 2.1% and 5.8%, respectively. 79, 78, 202, 11, and 7 patients received 2, 3, 4, 5, and 6 cycles of chemotherapy, respectively. The total cycles are 1297. Patients treated with 4–6 cycles of chemotherapy accounted for 58.4%.

### 2.3 Radiotherapy regime

All enrolled patients underwent ELEKTA linear accelerator 6MV‐X and treatment planning (ADAC pinnacle^3^ 7.4–8.2 treatment plan system) with 4 to 8 coplanar or non‐coplanar fields. Planning target volume (PTV) was determined by adding a 15 mm margin to the GTV. Three‐dimensional conformal radiation therapy (3D‐CRT) and intensity‐modulated radiation therapy (IMRT) were selected non‐randomly. The lung volume receiving ≥20 Gy (V_20_), the maximum dose of the spinal cord, and the average radiation dose of esophageal were ≤32%, 50 Gy, and ≤ 35 Gy, respectively. Within 1 week after the start of chemotherapy. The 3D‐CRT and IMRT of the whole group were 41 and 336 cases, respectively. The prescription dose for PTV was 60–70 Gy. The maximum prescription dose for the primary tumor was 76.5 Gy if normal tissues can tolerate it. The radiation dose of PTV was 30 to 76.5 Gy (median 60.0 Gy). Among them, <60.0 Gy, 60.0 to 76.5 Gy were 191 and 186 cases, respectively. Primary tumors were treated with late‐course accelerated hypofractionated radiotherapy (LAHR). The first course was 36–40 Gy at 2 Gy per once‐daily fraction, whereas LAHR was delivered in two fractions of 1.5 Gy each with an interval of 6–8 h each day. For distant metastases lesions, mainly treated by 3D‐CRT or 2D hypofractionated radiotherapy with 20–60 Gy at 3–10 Gy per once‐daily fraction. There were 265 cases of metastases lesions that underwent radiotherapy and 112 cases of non‐radiotherapy. The radiotherapy rate of metastases lesions was 70.3%.

All enrolled patients did the fiberoptic bronchoscopy and enhanced CT before treatment to evaluate primary tumors and regional lymph nodes in the chest, skull MRI to evaluate distant skull metastases, abdominal CT and bone scans, for patients with positive bone scans, implemented with MRI or enhanced CT, PET‐CT examinations were performed if necessary within 2 weeks before the start of treatment, and after the treatment, we repeated all the above tests except fiberoptic bronchoscopy and bone scan.

### 2.4 Evaluation of treatment‐related response and toxicity

The curative effect was evaluated according to the Response Evaluation Criteria in Solid Tumor System (RECIST) 1.0. The short‐term curative effect was evaluated 1 month after the end of treatment. Treatment‐related acute toxicity was scored with the National Cancer Institute's Common Terminology Criteria for Adverse Events, version 3.0. Blood routine and liver and kidney function should be tested before chemotherapy. Blood routine examinations should be done at least once a week during the course of treatment.

### 2.5 Follow‐up and statistical analyses

All the enrolled patients performed chest, abdominal enhanced CT, and skull MRI examinations every 3 months within the 2‐year treatment and every 6 months after the 2‐year treatment. Bone scans were performed every 6 months during the 2‐year treatment and once a year after the two‐year treatment. We define OS as the temporal duration that commences at the initiation of chemotherapy and terminates with the patient's death, regardless of the underlying cause. Similarly, PFS is delineated as the time span extending from the onset of chemotherapy to either the documented progression of the tumor or the patient's demise, attributable to any cause. All statistical analyses were performed using the Statistical Package for Social Sciences Version 15.0 (SPSS Inc, Chicago, USA). For survival analysis, the esteemed Kaplan–Meier method was employed. The significance of differences in proportions was assessed with the *χ* ^2^ test. The grouping and analysis of the GTV size and PTV radiation dose were conducted by establishing a threshold value utilizing the ROC curve methodology. The Cox proportional hazards model was used to perform multivariate analyses to assess the OS. Results were considered statistically significant when the two‐tailed *p*‐value was <0.05.

## 3 RESULTS

### 3.1 Survival time, GTV volume, and PTV cut‐off value

The last follow‐up was in January 2019. The total follow‐up time was 1–84 months, and the median follow‐up time was 13 months. The overall survival (OS) duration for the cohort of 377 patients ranged from 3 to 84 months, with a median survival time (MST) of 12.0 months. (95% CI, 11.2–12.8). Survival rates for 1‐, 2‐, 3‐, and 5‐year were 46.9%, 12.0%, 7.1%, and 3.2%, respectively. The ROC curve calculated the GTV volume cut‐off value in this study. Each point on the curve indicates the response of different GTV volume sizes to death. AUC = 0.643, Youden's index = 0.606 + 0.706–1 = 0.312, corresponding to a GTV volume of 150.443 cm^3^. Therefore, the cut‐off value was 150 cm^3^. According to this, there were 225 cases with GTV volume ≥ 150 cm^3^ and 152 cases with GTV volume < 150 cm^3^. The cut‐off value of the PTV radiotherapy dose is also calculated by the ROC curve. The points on the curve indicate the response of different PTV radiotherapy doses to survival. In particular, corresponding to a PTV radiotherapy dose volume of 62.957 Gy, we have AUC = 0.743, Youden's index = 0.882 + 0.556–1 = 0.438. Therefore, the cut‐off value was 63 Gy. There were 152 patients with GTV volume <150 cm^3^ which 11 survived and 141 died. And 225 patients with GTV volume ≥150 cm^3^ which 6 cases survived and 219 cases died.

### 3.2 Comparison of general clinical characteristics of IV NSCLC patients with different GTV volumes

In the comparison of general clinical characteristics of patients with GTV volume <150 cm^3^ and ≥150 cm^3^, there were statistical differences (*p* < 0.05) in N‐stage grouping, radiotherapy dose, chemotherapy cycle grouping, KPS score after treatment and short‐term efficiency as shown in Table 1.

**TABLE 1:** Comparison of general clinical characteristics of patients with NSCLC.

Characteristics	GTV <150 cm^3^ (*n* = 152)	GTV ≥150 cm^3^ (*n* = 225)	*p*‐values
Gender			
Male	102 (67.11)	180 (80.00)	0.088
Female	50 (32.89)	45 (20.00)	
Age			
<60	101 (66.45)	137 (60.89)	0.908
≥60	51 (33.55)	88 (39.11)	
Pathological type			
Squamous cell carcinoma	37 (24.34)	76 (33.78)	0.272
Non‐squamous cell carcinoma	115 (75.66)	149 (66.22)	
Primary tumor location			
Peripheral lung	87 (57.24)	107 (47.56)	0.164
Central lung	65 (42.76)	118 (52.44)	
T‐stage			
T1‐T2	75 (49.34)	66 (29.33)	0.483
T3‐T4	77 (50.66)	159 (70.67	
N‐stage			
N0–1	30 (19.74)	15 (6.67)	0.013
N2–3	122 (80.26)	210 (93.33)	
Number of metastatic organs			
1	87 (57.24)	104 (46.22)	0.053^a^
2	46 (30.26)	73 (32.45)	
3	15 (9.87)	39 (17.33)	
4	3 (1.97)	5 (2.22)	
5	1 (0.66)	4 (1.78)	
Radiotherapy Dose			
≥63 Gy	75 (49.34)	100 (44.44)	0.000
<63 Gy	77 (50.66)	125 (55.56)	
Chemotherapy cycles			
2	26 (17.11)	53 (23.56)	0.001^a^
3	30 (19.74)	48 (21.33)	
4	91 (59.86)	111 (49.33)	
5	3 (1.97)	8 (3.56)	
6	2 (1.32)	5 (2.22)	
KPS score before treatment			
=70	22 (14.47)	23 (10.22)	0.425
>70	130 (85.53)	202 (89.78)	
KPS score after treatment			
=70	32 (21.05)	109 (48.44)	0.029
>70	120 (78.95)	116 (51.56)	
Short‐term efficiency			
CR + PR	97 (63.82)	142 (63.11)	0.000
SD + PD	55 (36.18)	83 (36.89)	

Abbreviations: CR, complete remission; KPS, karnofsky performance status; PD, progressive disease; PR, partial remission; SD, stable disease.

^a^ Fish's exact test.

**FIGURE 1:**
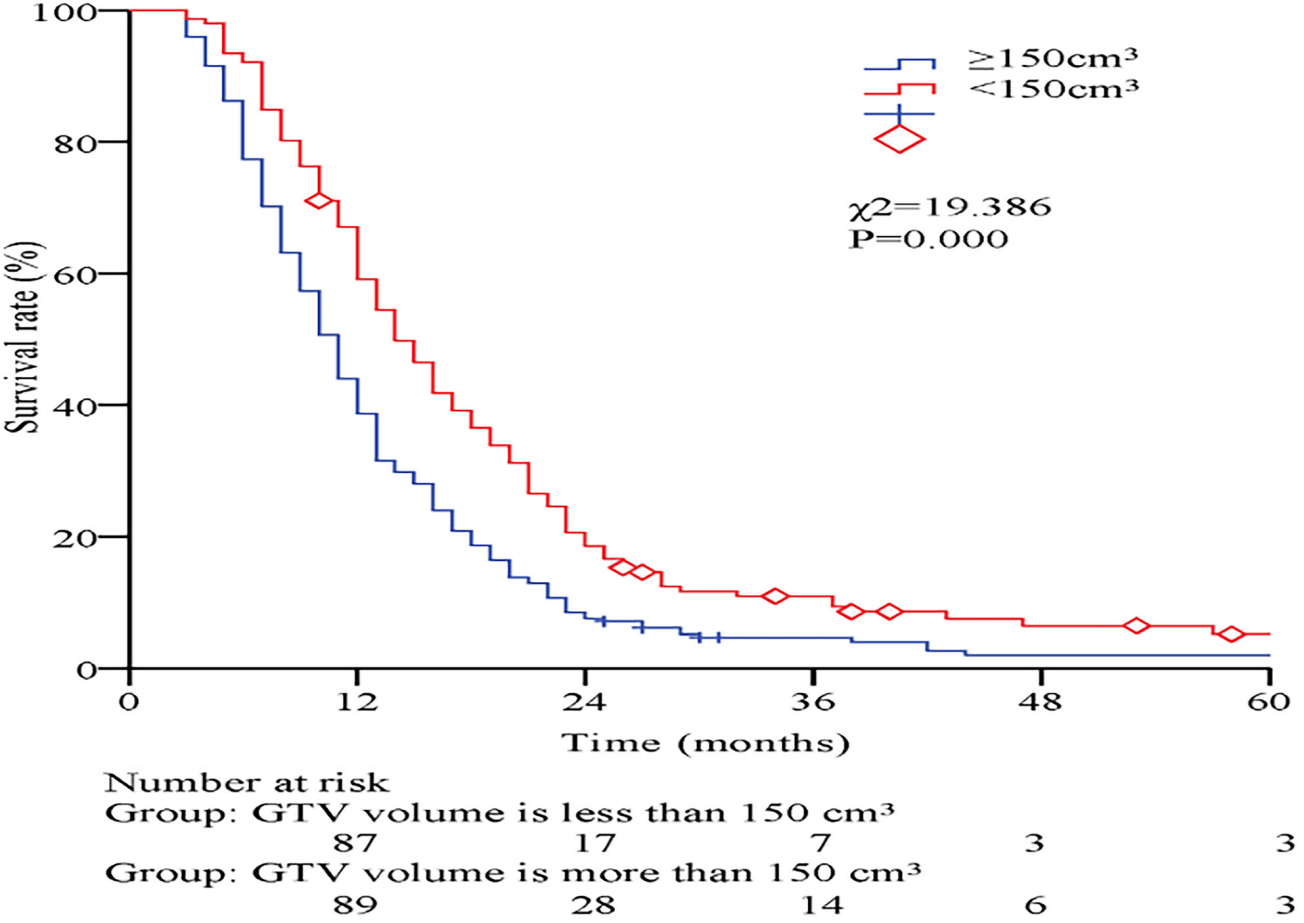
Survival curves for GTV volumes <150 cm^3^ and ≥150 cm^3^.

Survival analyses comparison between the two groups: GTV volume <150 cm^3^ and ≥150 cm^3^. The OS for 2 groups of 1, 2, 3, and 5‐year were 59.1% and 38.7%, 18.6% and 7.6%, 10.9% and 4.6%, 5.2% and 2.0%, respectively. The MST were 14.0 months (95% CI, 12.2–15.8) and 11.0 months (95% CI, 10.0–12.0), respectively. The difference between the two groups was statistically significant (*χ* ^2^ = 19.386, *p* = 0.000), as shown in Figure 1.

### 3.3 Survival time of patients with GTV <150 cm^3^ or GTV ≥150 cm^3^ between different groups

The analysis of survival outcomes for patients with a GTV of less than 150 cm^3^ revealed a significant survival advantage among those who achieved a KPS greater than 70 post‐treatment and presented with single organ metastases (*p* < 0.05). Conversely, for patients with a GTV equal to or exceeding 150 cm3, a survival benefit was observed in those who received a prescribed dose of at least 63 Gy to the PTV of the primary lesion, accompanied by 4–6 cycles of chemotherapy, resulting in CR or PR (*p* < 0.05). These findings are comprehensively presented in Table 2.

**TABLE 2:** Comparison of survival time of patients with GTV <150 cm^3^or ≥150 cm^3^.

Variables	Survival rates for GTV <150 cm^3^(%)					Survival rates for GTV ≥150 cm^3^ (%)				
	1‐year	2‐year	3‐year	5‐year	*p*‐values	1‐year	2‐year	3‐year	5‐year	*p*‐values
Gender										
Male	55.9	17.6	9.5	4.8	0.399	38.9	6.7	3.7	1.5	0.683
Female	65.6	20.5	14.1	5.9		37.8	11.1	8.3	4.2	
Age										
<60	58.2	16.1	9.5	4.1	0.666	37.2	8.0	3.7	2.5	0.833
≥60	60.8	23.5	13.7	7.4		40.9	6.8	5.7	1.4	
KPS before treatment										
≤70	36.4	22.7	18.2	6.8	0.590	34.8	4.3	0.0	0.0	0.354
>70	63.0	17.9	9.6	5.1		39.1	7.9	5.2	2.2	
KPS after treatment										
≤70	39.8	13.3	8.9	4.4	0.034	38.5	8.3	5.5	4.1	0.741
>70	64.2	20.0	11.5	5.5		38.8	6.9	3.7	0.0	
Pathological type										
Squamous cell carcinoma	54.1	13.5	13.5	0.0	0.285	42.1	5.3	5.3	1.8	0.899
Non‐squamous cell carcinoma	60.7	20.2	10.2	6.6		36.9	8.7	4.1	2.1	
Radiotherapy dosage										
<63 Gy	54.5	13.0	4.5	1.5	0.110	33.6	2.4	2.4	0.0	0.002
≥63 Gy	63.8	24.4	17.5	8.9		45.0	14.0	7.6	4.6	
Metastatic organs										
Single‐organ metastases	68.8	22.2	15.0	4.8	0.028	41.3	9.6	6.4	3.8	0.314
Multi‐organ metastases	46.2	13.8	5.4	5.4		36.4	5.8	3.0	0.0	
T‐Stage										
T1–T2	58.7	14.7	8.0	5.0	0.399	45.5	7.6	5.7	1.9	0.542
T3–T4	59.5	22.5	13.9	3.9		35.8	7.5	4.2	2.1	
N‐Stage										
N0–N1	66.3	31.4	20.0	1.0	0.081	46.7	6.7	6.7	6.7	0.441
N2–N3	57.4	15.6	8.8	4.2		38.1	7.6	4.5	1.5	
Chemotherapy cycles										
2–3 cycles	55.4	17.9	10.7	6.4	0.678	25.7	3.0	1.0	0.0	0.000
4–6 cycles	61.3	19.0	11.1	4.9		49.2	11.3	7.7	3.8	
Short‐term efficiency										
CR + PR	66.0	19.6	12.0	6.8	0.157	45.1	10.6	6.7	3.3	0.000
SD + PD	46.8	16.8	9.4	2.3		27.7	2.4	1.2	0.0	

Abbreviations: CR, complete remission; KPS, karnofsky performance status; PD, progressive disease; PR, partial remission; SD, stable disease.

### 3.4 Comparison of survival for the same volume of primary tumors between different radiotherapy and chemotherapy treatments regime

Fifty‐six patients received 2 to 3 cycles of chemotherapy in GTV <150 cm^3^. The 1, 2, 3, and 5‐year survival rates of 16 patients receiving radiation doses of ≥63 Gy and 40 patients <63 Gy were 56.3% and 55.0%, 31.3% and 12.5%, 25.0% and 5.0%, 16.7% and 2.5%, respectively. Their MSTs being 13 (95% CI, 9.0–16.9) and 13 (95% CI, 10.9–15.0) months, respectively, (*χ* ^2^ = 2.456, *p* = 0.117). Ninty‐six patients received 4 to 6 cycles of chemotherapy in GTV <150 cm^3^. The 1, 2, 3, and 5‐year survival rates of 59 patients receiving radiation doses of ≥63 Gy or 37 patients <63 Gy were 65.9% and 54.1%, 22.5% and 13.5%, 15.4% and 3.6%, 7.7% and 0.0%, respectively. Their MSTs being 17 (95% CI, 14.5–19.4) and 13 (95% CI, 9.7–16.4) months, respectively, (χ^2^ = 3.837, *p* = 0.050).

101 patients with 2 to 3 cycles of chemotherapy in GTV ≥150 cm^3^. The 1, 2, 3, and 5‐year survival rates of 35 patients receiving radiation doses of ≥63 Gy and 66 patients <63 Gy were 34.3% and 21.2%, 8.6% and 0.0%, 2.9% and 0.0%, 0.0% and 0.0%, respectively, their MSTs being 10 (95% CI, 6.7–11.3) and 8 (95% CI, 6.4–9.6) months, respectively, (*χ* ^2^ = 4.620, *p* = 0.032). One hundred and twenty‐four patients received 4 to 6 cycles of chemotherapy in GTV ≥150 cm^3^, the 1, 2, 3, and 5‐year survival rates of 65 patients receiving radiation doses of ≥63 Gy or 59 patients <63 Gy were 50.8% and 47.5%, 16.9% and 5.1%, 10.3% and 5.1%, 7.7% and 0.0%, respectively. Their MSTs being 13 (95% CI, 11.0–14.9) and 12 (95% CI, 10.3–13.7) months, (*χ* ^2^ = 2.384, *p* = 0.092).

### 3.5 The survival comparison for GTV volume <150 cm^3^ and ≥150 cm^3^

Among patients with 2–3 cycles of chemotherapy and PTV ≥63 Gy, the 1, 2, 3, and 5‐year survival rates of patients with GTV <150 cm^3^ and GTV ≥150 cm^3^ were 56.3% and 32.4%, 31.3% and 8.8%, 25.0% and 2.9%, 16.7% and 0.0%, respectively. The MST was 13.0 months and 9.0 months, respectively. There was a significant difference between the two groups (*χ* ^2^ = 5.868, *p* = 0.015).

Among patients with 2–3 cycles of chemotherapy and PTV <63 Gy, the 1, 2, 3, and 5‐year survival rates of patients with GTV <150 cm^3^ and GTV ≥150 cm^3^ were 55.0% and 22.4%, 12.5% and 0.0%, 5.0% and 0.0%, 2.5% and 0.0%, respectively. The MSTs were 13 months and 8 months, respectively. There was a significant difference between the two groups (*χ* ^2^ = 16.413, *p* = 0.000).

Among patients with 4–6 cycles of chemotherapy and PTV ≥63 Gy, the 1, 2, 3, and 5‐year survival rates of patients with GTV <150 cm^3^ and GTV ≥150 cm^3^ were 65.9% and 50.8%, 22.5% and 16.9%, 17.3% and 10.3%, 7.7% and 7.7%, respectively. The MSTs were 17 months and 13 months, respectively, (*χ* ^2^ = 2.358, *p* = 0.125).

Among patients with 4–6 cycles of chemotherapy and PTV <63 Gy, the 1, 2, 3, and 5‐year survival rates of patients with GTV <150 cm^3^ and GTV ≥150 cm^3^ were 54.1% and 47.5%, 13.5% and 5.1%, 3.6% and 5.1%, 0.0% and 0.0%, respectively. The MSTs were 13.0 months and 12.0 months, respectively, (χ^2^ = 1.121, *p* = 0.290).

### 3.6 Multivariate Cox regression analyses for factors affecting prognostic of stage IV NSCLC

Through multivariate Cox regression analyses, it was found that good prognostic factors for patients with stage IV NSCLC are: Peripheral lung cancer, radiation dose ≥63 Gy, GTV <150 cm^3^, 4–6 cycles of chemotherapy, CR + PR. Table 3.

**TABLE 3:** Multivariate analyses of different variables and patient prognosis for stage IV NSCLC.

Variables	HR	95% confidence intervals (CIs)		χ^2^	*p*‐values
		Lower limit	Upper limit		
Gender (female vs. male)	0.794	0.620	1.018	3.312	0.069
Age (≥ 60 vs. <60)	0.879	0.706	1.095	1.319	0.251
KPS before treatment (>70 vs. =70)	0.811	0.586	1.122	1.602	0.206
KPS after treatment (>70 vs. =70)	0.924	0.713	1.196	0.363	0.547
Pathological type (non‐SCC vs. SCC)	1.045	0.813	1.344	0.120	0.729
Primary tumor location (peripheral vs. central)	0.802	0.650	0.991	4.180	0.041
Radiation dose (<63 Gy vs. ≥63 Gy)	1.455	1.172	1.805	11.578	0.001
Metastatic organs (single‐organ vs. multi‐organ)	0.829	0.672	1.023	3.043	0.081
T stage (T3‐4 vs. T1‐T2)	0.976	0.779	1.222	0.046	0.830
N stage (N2‐N3 vs. N0‐N1)	1.236	0.876	1.746	1.455	0.228
GTV volume (<150 cm^3^ vs. ≥150 cm^3^)	0.648	0.520	0.809	14.802	0.000
Chemotherapy cycles (4–6 vs. 2–3)	0.760	0.612	0.944	6.155	0.013
Metastasis radiotherapy (no vs. yes)	0.984	0.774	1.250	0.018	0.892
Overall efficiency (SD + PD vs. CR + PR)	1.447	1.160	1.804	0.757	0.001

Abbreviations: CI, confidence interval; CR, complete remission; HR, hazard ratio; KPS, karnofsky performance status; non‐SCC, non‐squamous cell carcinoma; PD, progressive disease; PR, partial remission; SCC, squamous cell carcinoma; SD, stable disease.

### 3.7 Toxic side effects during concurrent chemoradiotherapy for patients with stage IV NSCLC

Most of the side effects during the concurrent chemoradiotherapy are grade 0–2 white blood cell decline, grade 0–2 neutrophil absolute value decrease, and hemoglobin and platelet decrease. Whereas one case is grade 5 in blood platelets. Grade 1–2 gastrointestinal reactions, grade 0–1 radiation pneumonia, whereas two cases of grade 5 and grade 0–2 radiation esophagitis occurred. The detailed information is shown in Table 4.

**TABLE 4:** Toxicity during concurrent radiotherapy and chemotherapy.

Toxic reaction	Grade 0–1	Grade 2	Grade 3	Grade 4	Grade 5
Vomiting	194 (51.46)	153 (40.58)	30 (7.96)	‐	‐
Diarrhea	353 (93.64)	19 (5.03)	4 (1.06)	1 (0.27)	‐
White blood cells	77 (20.42)	138 (36.61)	110 (29.18)	52 (13.79)	‐
Neutrophil absolute value	166 (44.03)	93 (24.67)	71 (18.83)	47 (12.47)	‐
Blood platelet	288 (76.39)	40 (10.61)	33 (8.75)	15 (3.98)	1 (0.27)
Hemoglobin	298 (79.05)	55 (14.59)	18 (4.77)	6 (1.59)	‐
Radiation pneumonia	350 (92.83)	20 (5.30)	5 (1.33)	‐	2 (0.54)
Radiation esophagitis	214 (56.76)	39 (36.87)	24 (6.37)	‐	‐

### 3.8 Analyses of patients' death reason

After this study, 37.59% of patients with GTV volume <150 cm^3^ died due to local progression and 52.50% of patients with GTV volume ≥150 cm^3^ died due to local progression. There was a significant difference between the two groups (*p* = 0.000). The detailed information is shown in Table 5.

**TABLE 5:** Analyses of Patients' Death Reason.

Condition	Death reasons	GTV volume <150 cm^3^	GTV volume ≥150 cm^3^	*p*‐values
Death	Local progression	53 (37.59)	115 (52.50)	0.000
	Distant metastases	43 (30.50)	44 (20.07)	0.027
	Local progression and distant metastases	21 (14.89)	34 (15.52)	0.004
	Blood or bone marrow suppression	1 (0.71)	1 (0.46)	0.083^a^
	Radiation pneumonia	2 (1.42)	0 (0.00)	0.083^a^
	Lungs and upper respiratory tract	2 (1.42)	4 (1.84)	0.239^a^
	General cardiovascular disease	0 (0.00)	1 (0.46)	1.000^a^
	Hemoptysis	0 (0.00)	2 (0.92)	0.083^a^
	Infection	3 (2.13)	5 (2.30)	0.137^a^
	Death of unknown cause	16 (11.34)	13 (5.93)	0.305^a^

^a^ Fish's exact test.

## 4 DISCUSSION

The purpose of this study was to investigate the effect of different tumor volumes on patients' survival with stage IV NSCLC undergoing concurrent chemoradiotherapy. Previous studies have investigated the effect of primary tumor volume on the survival of patients with stage I–III NSCLC and found that patients with small‐volume tumor survive better than patients with large‐volume tumor.^16^, ^17^, ^18^ In recent years, studies have found that almost 50% of patients with stage IV NSCLC have recurrence cancer in the initial affected area. Local control of the primary tumor is related to OS.^21^, ^22^, ^23^, ^24^, ^25^ Su et al.^12^ performed a multi‐center prospective Phase II clinical study and found that primary tumor volume was related to OS of patients with stage IV NSCLC by multivariate analyses. This study is consistent with previous findings that primary tumor volume affects survival in patients with stage IV NSCLC.

Fairchild^26^ conducted a systematic retrospective analyses of 13 randomized controlled studies from 1985 to 2005 and concluded that increasing the local dose of primary tumors in the chest not only improved symptoms but also significantly prolonged the MST and 1–2 year survival rate. They proposed the necessity and importance of local radiotherapy for primary tumors in some cases. Therefore, local control of primary tumors is becoming increasingly important. The ideal treatment model for stage IV NSCLC is to control both primary tumors and metastatic lesions. Concurrent chemoradiotherapy conforms to this model.

Li et al.^27^ performed a meta‐analysis and found that the survival time of patients with stage IV NSCLC enduring primary tumor radiotherapy was better than that of patients without primary tumor radiotherapy. Female, no lymph node metastases, and adenocarcinoma were good prognostic factors in patients with stage IV NSCLC. However, prospective clinical studies are still needed to be done to prove it.

Maria et al.^28^ analyzed 161 stage I‐III NSCLC patients, and the results showed that the MST and progression‐free survival (PFS) of those with small tumor volume was significantly higher than those with large tumor volume. They also believed that patients undergoing future clinical trials of radiation therapy should use tumor volume for stratified analyses. Tumor volume and radiation dose have a direct relationship with patients' prognosis and disease control. Su et al.^29^ conducted a prospective study and found that combined with radiotherapy dosage ≥63 Gy on the basis of effective systemic chemotherapy may prolong the survival of patients with non‐oligo metastatic stage IV NSCLC. Primary tumor volume may be used as the criterion for determining radiation dose.

Ouyang et al.^17^ performed the stratified analyses of different chemotherapy regimens for patients with stage IV NSCLC and found that patients with 4–6 cycles of systemic chemotherapy combined with primary tumor radiotherapy ≥63 Gy have higher survival rates than other treatment models (4–6 cycles with primary tumor radiotherapy <63 Gy, primary tumor radiotherapy for 2–3 cycles ≥63 Gy or <63 Gy).

Under the same conditions of primary tumor volume, the GTV <150 cm^3^, performing 2 to 3 cycles of chemotherapy concurrent 3D radiotherapy on primary tumors. There was no significant difference in survival rate between ≥63 Gy and <63 Gy. When performing the same period of 4 to 6 cycles of chemotherapy concurrent 3D radiotherapy, patients of irradiation dosage ≥63 Gy has a significant difference in survival compared with patients of irradiation dosage <63 Gy. It can be found that high‐dose radiotherapy can improve the survival rate for patients with smaller volumes when chemotherapy intensity is sufficient. In the group of patients with GTV ≥150 cm^3^, it can be found that high‐dose radiotherapy can improve the survival of patients when chemotherapy intensity is insufficient. However, the radiation dose of the primary tumor has no significant difference in the survival of patients when the intensity of chemotherapy is sufficient.

Compared to stage IV NSCLC patients with GTV <150 cm^3^ and GTV ≥150 cm^3^, patients with smaller volumes survived better than patients with larger volumes when they underwent 2–3 cycles of chemotherapy. Patients with smaller volumes have high survival rate than patients with larger volumes when chemotherapy intensity is insufficient. Compared GTV <150 cm^3^ with GTV ≥150 cm^3^ patients undergoing 4–6 cycles of chemotherapy, there was no significant difference in the comparison of survival time. It may be considered that increasing the intensity of chemotherapy can improve the survival rate of patients with larger tumor volumes.

37.59% of patients with GTV <150 cm^3^ died due to local progression and 52.50% of patients with GTV ≥150 cm^3^. The mortality caused by the local progression of patients with GTV ≥150 cm^3^ increased significantly. Therefore, the prognosis is poor for patients with large tumor volumes. Multivariate analyses show that peripheral lung cancer, radiation dose ≥63 Gy, GTV <150 cm^3^, 4–6 cycles of chemotherapy, and CR + PR are good prognostic factors for patients with stage IV NSCLC.

For patients with stage IV NSCLC, palliative radiation therapy was mainly used to relieve the symptoms of cough, hemoptysis, chest pain, and dyspnea caused by primary tumors.^24^, ^30^, ^31^, ^32^ It can also relieve intrathoracic bronchial and vascular compression.^33^ With the development of three‐dimensional radiotherapy, we have increased the radiation dose of tumor tissues and reduced radiation damage to normal tissues. It becomes possible for patients with stage IV NSCLC to undergo radical radiotherapy. According to the 2019 NCCN Guidelines for Lung Cancer, oligometastatic stage IV NSCLC metastases can be treated with stereotactic ablation for radical radiotherapy. Stereotactic radiotherapy is most commonly used for tumors which is smaller than 5 cm. As for more selective isolated tumors, radiotherapy can be safely performed if the dose of normal tissue is guaranteed to be tolerable.^34^, ^35^

Petrelli et al.^36^ found that patients with oligometastatic stage IV NSCLC can prolong OS and PFS when using primary tumor radical radiation therapy. This study found systemic chemotherapy and primary tumor radical radiotherapy can prolong the survival of patients with small volume groups. In patients with larger tumor volumes group, primary tumor radical radiotherapy can prolong the survival of patients when chemotherapy intensity is insufficient. Therefore, primary tumor radiotherapy is very important. Primary tumor volume, radiation dose, and the cycle of chemotherapy have a direct relationship with patients' prognosis.

Another issue with concurrent chemoradiotherapy for stage IV NSCLC involves its potential toxicity. One patient in the current study died of thrombocytopenia, but the mortality rate of chemotherapy alone for advanced NSCLC was between 1.5% and 3.0%.^10^, ^37^ Compared with locally advanced NSCLC concurrent radiotherapy,^38^, ^39^ there was no increase in the incidence of radiation pneumonia and esophagitis in this study. Treatment‐related hematological or non‐hematological toxicity in current studies are acceptable.

## 5 CONCLUSION

Patients with GTV <150 cm^3^ have longer survival time than patients with GTV ≥150 cm^3^. Patients with GTV <150 cm^3^ have a higher survival rate than those with ≥150 cm^3^ when they undergo 2 to 3 cycles of chemotherapy concurrent with 3D radiotherapy. When performing the 4 to 6 cycles of chemotherapy concurrent 3D radiotherapy, there was no significant difference in survival rate between GTV <150 cm^3^ and ≥150 cm^3^.

## VOLUNTARY PARTICIPATION

Crucially, our informed consent process emphasizes that participation in the study is entirely voluntary. Participants are made aware that they have the right to decline participation, withdraw from the study at any time without penalty or loss of benefits to which they are otherwise entitled, and to seek clarification or additional information if needed. By obtaining written informed consent, we affirm that each participant has, after careful consideration, voluntarily agreed to participate in the study. This agreement serves as a testament to their autonomy and underscores their active role in shaping the course of research that may ultimately contribute to scientific knowledge and advance medical practice.

## AUTHOR CONTRIBUTIONS

**Xiaxia Chen:** Data curation (equal); resources (equal); software (equal); writing – original draft (equal). **Wei Zhang:** Data curation (equal); investigation (equal); resources (equal); software (equal); writing – original draft (equal). **Lan Luo:** Resources (equal); software (equal). **Shimei Fu:** Resources (equal). **Dongdong Cao:** Resources (equal). **Shengfa Su:** Software (equal); supervision (equal). **Qingsong Li:** Resources (equal). **Wengang Yang:** Resources (equal). **Yichao Geng:** Resources (equal); software (equal). **Bing Lu:** Resources (equal); software (equal). **Weiwei Ouyang:** Data curation (lead); resources (lead); software (lead); supervision (lead); writing – review and editing (lead).

## CONFLICT OF INTEREST STATEMENT

None.

## ETHICS STATEMENT

At our institution, we hold the highest regard for ethical principles and standards when conducting research involving human participants or utilizing patient material. It is our unwavering commitment to ensure that every aspect of our research endeavors adheres to the strictest ethical guidelines, safeguarding the rights, safety, and well‐being of all individuals involved.

## CONSENT

We would like to emphatically clarify that, in all studies involving human participants or the use of patient material, we have obtained written informed consent from each and every participant prior to their enrollment in the research. The informed consent process is designed to provide participants with a comprehensive understanding of the study, including its nature, purpose, methodology, anticipated duration, potential risks and benefits, as well as any alternative treatment options or procedures available to them. We strive to present this information in a clear, concise, and understandable manner, taking into account the varying literacy levels and comprehension abilities of our participants. Moreover, we ensure that the consent process is conducted in a language that the participant fully understands, either their native language or through the use of professional interpreters when necessary. This ensures that no participant is excluded from making an informed decision based solely on language barriers.

## Data Availability

Data and supplementary material for this study are available.
